# Supplementary material for: Single-Cell RNAseq Resolve the Potential Effects of LanCL1 Gene in the Mouse Testis
Source: Cells. 2022 Dec 19;11(24):4135. doi: 10.3390/cells11244135 (PMC9777014; doi:10.3390/cells11244135)
Supplement: Supplementary file 1 [file cells-11-04135-s001.zip › Table S1.pdf]

Antibodies	Source	Identifier
Rabbit anti-Lancl 1	Thermo Scientific	Cat# PA5-57107; AB_2643270
Rabbit anti-β-actin	Cell Signaling Technology	Cat#4970S; AB_2223172
Donkey anti-rabbit Alexa555	Thermo Scientific	Cat#31572; RRID: AB_162543
DAPI	Thermo Scientific	Cat#D3571
Testosterone Assay K	R&D Systems	Cat#KGE010
RNeasy Mini Kit	Qiagen	Cat#74104
