# Supplementary figures and images for: Single-Cell RNAseq Resolve the Potential Effects of LanCL1 Gene in the Mouse Testis
Source: Cells. 2022 Dec 19;11(24):4135. doi: 10.3390/cells11244135 (PMC9777014; doi:10.3390/cells11244135)

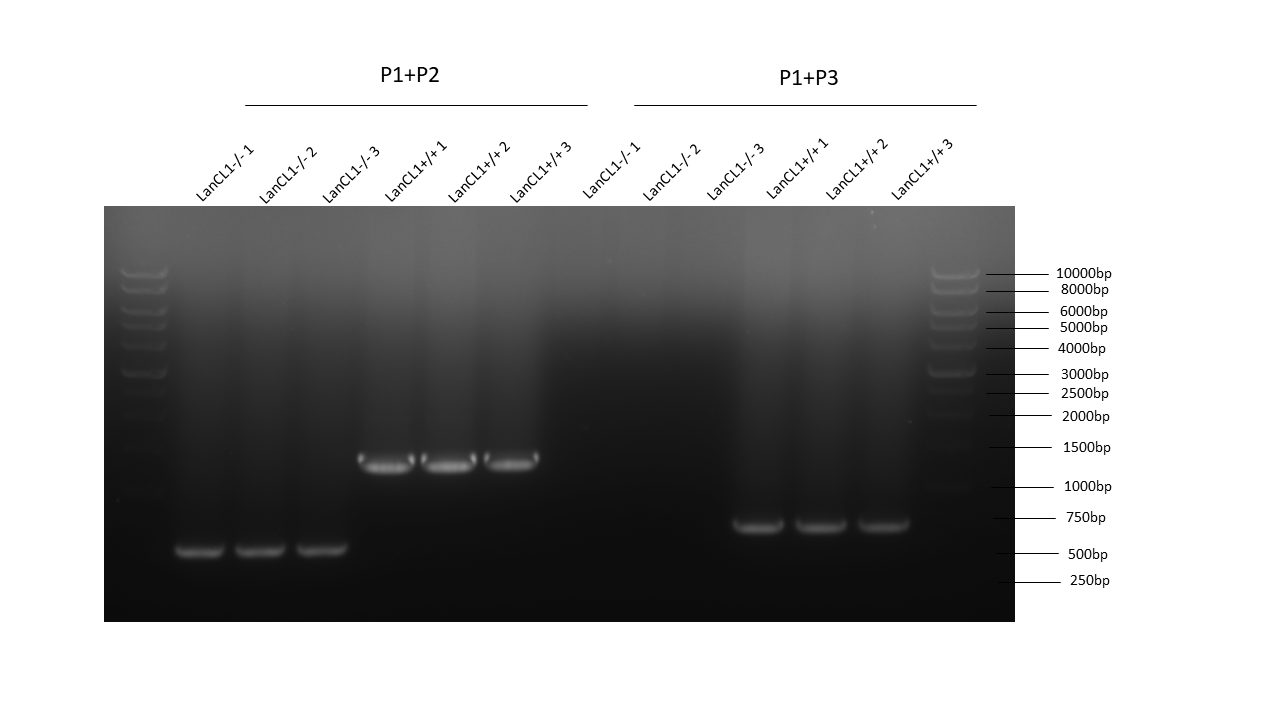

Supplement: Supplementary file 1 [file cells-11-04135-s001.zip › Supplementary Figure S1.tif]
